# Increasing the Consumption of Environmentally Friendly Foods in a University Dining Hall Using Menu Item Placement

**DOI:** 10.3390/nu15183873

**Published:** 2023-09-06

**Authors:** Cinzia Franchini, Carole Bartolotto, Francesca Scazzina, Catherine L. Carpenter, Wendelin Slusser

**Affiliations:** 1 Human Nutrition Unit, Department of Food and Drug, University of Parma, 43121 Parma, Italy; cinzia.franchini@unipr.it (C.F.); francesca.scazzina@unipr.it (F.S.); 2 UCLA Housing, University of California at Los Angeles (UCLA), Los Angeles, CA 90095, USA; cbartolotto@ha.ucla.edu; 3 Division of Clinical Nutrition, Center for Human Nutrition, David Geffen School of Medicine, University of California at Los Angeles (UCLA), Los Angeles, CA 90095, USA; 4 School of Nursing, University of California at Los Angeles (UCLA), Los Angeles, CA 90095, USA; 5 Department of Pediatrics, David Geffen School of Medicine, University of California at Los Angeles (UCLA), Los Angeles, CA 90095, USA; wslusser@conet.ucla.edu; 6 Department of Community Health Sciences, Fielding School of Public Health, University of California at Los Angeles (UCLA), Los Angeles, CA 90095, USA; 7 Semel Healthy Campus Initiative Center, University of California at Los Angeles (UCLA), Los Angeles, CA 90095, USA

**Keywords:** university students, canteen, food choices, menu, nudging, climate change, carbon footprint, environmental sustainability

## Abstract

Creating a decision-making environment that promotes sustainable food choices is a priority for both the individual and society. This study aimed at encouraging plant-based menu choices by re-ordering the menu according to the carbon footprint values. The project was conducted in a grab-and-go eatery at a large United States public university, where students could order their meals choosing among different menu options that were customizable with various ingredients. The order of menu ingredients was changed twice: for five weeks, from the most to the least impactful in terms of carbon footprint; subsequently, for another five weeks the order was reversed. At both times, all sales data were recorded. A total of 279,219 and 288,527 items were selected, respectively, during the first and the second intervention. A significant association was found between menu re-ordering and customers’ choices for almost all food categories considered. Overall, despite beef choices not changing, results showed that students were more likely to choose low-carbon options when these were placed at the beginning, emphasizing that food selections were impacted by ingredient placement on the menu list. These findings highlight the need for a multi-level strategy focused on raising students’ awareness of the environmental impact of animal-based foods, particularly beef.

## 1. Introduction

The effects of global warming are increasingly visible, such as higher ambient temperatures, changing rainfall, rising ocean levels, and an overall increase in the frequency and severity of meteorological events [1]. The latest 2019 estimates report exponential growth in food system-related greenhouse gas (GHG) emissions over the past three decades, accounting for 31% of all global human-related emissions that reflect current climate change [2,3]. Food systems represent one of the main causes of climate change and, at the same time, are negatively affected by it [4]. Interruption of the food production chain and declining crop yields are results of the current alarming global weather situation that, if not resolved, will negatively influence the availability of safe and nourishing foods, resulting in a greater risk of poverty, malnutrition, and hunger across the world [4,5,6,7].

Depending on the source, foods require specific production processes that affect environmental resources differently. The environmental pressure of different food groups might be expressed through different ecological implications (e.g., GHG emissions, cropland and water use, nitrogen and phosphorus application) [8,9,10]. Taking GHG emissions into consideration, the abbreviation “carbon footprint” is often used as a summary indicator to quantify the environmental impact—expressed in terms of kilograms of carbon dioxide equivalent emissions (CO_2_ eq)—associated with the production of a commodity or service considering the entire life cycle [11,12]. In particular, the carbon footprint of animal-source food is much higher than plant-based food, whose environmental impact, in comparison, appears rather negligible. In addition, the GHG emissions from foods of animal origin are highly variable, for example, depending on the animal and how it is raised and processed [8,9,13]. Poultry and animal by-products, such as eggs, milk, and yogurt, are the least impactful land-based animal products in terms of carbon footprint. The highest GHG emissions are associated with the food chain represented by, for example, red meat, cheese, processed meat, and fish, in descending order [14]. In particular, beef has four times the carbon footprint of chicken, pork, or fish [13]. However, GHG emissions of seafood products vary greatly depending on fishing methods [15], thus, the transition to sustainable fisheries and aquaculture is a priority of General Fisheries Commission for the Mediterranean (GFCM) 2030 Strategy [16].

Considering the crucial role of food systems in combating climate change and related problems, several policies need to be implemented to address this issue of global importance [4]. The quantity and typology of foods we choose to eat on a daily basis impacts our health and the planet’s well-being [9,17,18]. Therefore, people’s dietary choices have a major impact on the transformation of our food system [19]. In this context, one of the most important and demanding challenges of our century is shifting people’s eating habits toward healthy and sustainable diets [20,21] that primarily include a large and varied consumption of fruits, vegetables, and other plant-based foods, and a low intake of animal-based products [22]. Considering that the production of animal-source products has increased by more than 60% worldwide in recent decades, choosing foods with lower-carbon-footprint values becomes especially important [23]. To achieve this goal, comprehensive policies that include a multi-strategy approach need to be implemented. Among these, creating a decision-making environment that promotes sustainable food choices is a priority for both the individual and society [4,24]. Positively influencing people’s choices through nudge intervention, such as eliminating, adding to, or modifying decision factors, is an approach to improving people’s health [24] that can be easily deployed in food services, including university cafeterias [25,26].

The life period between adolescence and young adulthood represents a delicate time of transition and change in habits [27,28], offering opportunities to promote and support healthy eating behaviors. Among young adults, college students often eat their meals in dining halls. For this reason, dining services represent a strategic venue for the implementation of interventions to ensure healthy and sustainable diets for young populations, with the goal of preventing long-term health risks while simultaneously reducing the pressure and impact of food systems on the environment [29,30,31]. One of the most light-touch and low-cost nudge interventions is to change the layout of the menu by changing dishes’ descriptions or, more simply, rearranging their positions [32,33], by taking advantage of people’s innate gaze-motion and memory tendencies [34]. Furthermore, a recent systematic review identified menu restructuring as the most effective intervention to shift users’ choices toward more sustainable diets, particularly by placing green options at the top of the list to enhance their visibility and likelihood of being chosen [35].

To determine whether the order of menu items could influence sustainability, this intervention study aimed to increase climate-friendlier dietary choices by reordering customizable menu items according to their carbon footprint values (CO_2_ eq).

## 2. Materials and Methods

### 2.1. Setting and Study Design

This pilot study was conducted during the fall quarter 2021 at the University of California Los Angeles (UCLA) within the UCLA dining hall, The Study at Hedrick. UCLA Dining Services provides breakfast, lunch, and dinner to the UCLA community, serving a total of around 6.5 million meals per year, representing one of the biggest independent catering services in the United States. The Study at Hedrick can be attended by students on a meal plan, which includes a prepaid number of meals and the ability to choose from a variety of foods and beverages without being influenced by price. On-site students order their meals using an iPad containing all menu items stationed on a pedestal at the entrance of the grab-and-go eatery. Once they order on the iPad, users move to the counter of the food pickup area to retrieve their meal. A screen listing all of the menu items in alphabetical order was located near the counter area, but that menu was not visible to students when they entered the dining hall. The on-site menu display included different menu options (i.e., pizza, salads, skillets, bagels, and sandwiches) that were customizable by choosing from different food items. Pictures of how the menu appeared on the iPad are provided in Supplementary Materials S1. The menu configuration was in use before the re-ordering intervention.

**Figure 1:**
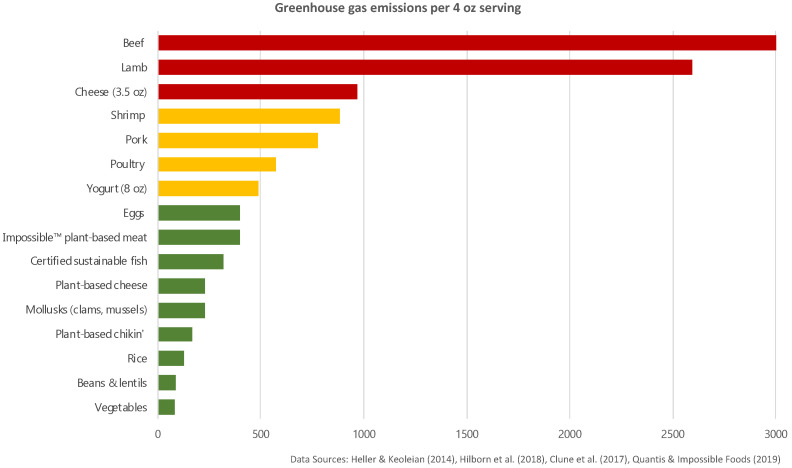
Carbon Footprint Scorecard developed by UCLA.

Firstly, each customizable item’s carbon footprint (g CO_2_ equivalent) was determined following the methodology described in Malan et al. [36]. Briefly, we used the Carbon Footprint Scorecard [36] developed by UCLA based on estimates of GHG (Greenhouse Gas) emissions reported in the following four literature references: Heller & Keoleian, 2014 [37], Clune et al., 2017 [13], Hilborn et al., 2018 [38], and Quantis & Impossible Foods (Khan et al., 2019) [39] (Figure 1).

In addition, based on the Planetary Health Diet recommendations provided by the Eat-Lancet Commission [22], a daily value (DV) of the dietary carbon footprint was calculated and the food categories were ranked according to their contribution to it: low (0–25% of the DV), medium (26–50% of the DV), and high (>50% of the DV). This classification was made by considering the GHG emissions for one serving (i.e., 4 ounces) [36]. For items composed of multiple ingredients (e.g., herb cream cheese), the level of carbon footprint was estimated by considering the main component of the recipe. According to the study of Malan and colleagues [36] mentioned above, this classification method was followed to facilitate future long-term implementation of this intervention. Finally, most fish purchased and served at UCLA dining is certified as sustainable by the Seafood Watch of Monterey Bay Aquarium [40] and thus falls within low-carbon-footprint food categories.

**Figure 2:**
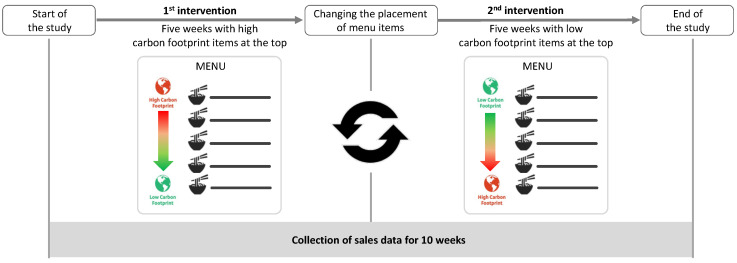
Diagram of the study design.

Normally, the customizable items of different menu options are in alphabetical order, but during this study, the order was modified twice (Figure 2). For the first five weeks (1st intervention), the customizable menu items and add-ons were listed in descending order from the highest to lowest carbon footprint. Subsequently, for the following five weeks (2nd intervention), the order was reversed, and the items were sorted in ascending order, from the food with the lowest at the top of the list to the one with the highest carbon footprint at the bottom.

To further clarify, during the first intervention, at the top of the menu were high-carbon-footprint items or medium-carbon-footprint items in case there were no high-carbon-footprint foods for that menu type. In the second intervention, the menu always began with low-carbon food options. Considering the type of intervention, which modified the position of food options on the menu without excluding any and did not involve collecting information from diners, the UCLA Institutional Review Board determined the study was “Exempt” from Informed Consent, and it was not deemed necessary to inform students about the study.

### 2.2. Data Collection

During both the first and second interventions, the food choices of all customers who dined at The Study at Hedrick were collected for five weeks by evaluating the sales data, which were recorded through the centralized MyMicros web-delivered reporting platform and exported to an Excel worksheet as the total quantity sold for each menu item during the two intervention periods.

### 2.3. Data Analysis

Sales data for each customizable item were categorized into 13 unique foods with different carbon footprint levels: high (beef and cheese), medium (pork and poultry), and low (eggs, plant-based meat, certified sustainable fish, plant-based cheese, cereals, legumes, nuts, fruit, and vegetables). Proportions of sales within each food group were compared according to the two intervention conditions. We conducted two sets of analyses: summarizing sales into the 13 categories described above and summarizing the sales into two carbon footprint categories (low- versus medium- or high-carbon footprint). Binomial proportion tests were applied for both analyses to explore possible significant differences in sales distribution during the two periods considered. Our null hypothesis was that ordering menu items by carbon footprint made no difference in purchasing. All statistical analyses were performed through the IBM SPSS Statistics for Macintosh, version 28.0 (Armonk, NY, USA: IBM Corp), *p*-value less than 0.05 was considered statistically significant.

## 3. Results

A total of 279,219 and 288,527 items were selected, respectively, during the first and the second interventions. As shown in Table 1, considering the diverse food categories, some food typologies were selected more often during both interventions; the most chosen food categories were cheese (27% and 25%), pork (18% and 17%), and poultry (15% and 14%) for products with high- to medium-carbon footprint (66% and 58%), and fruit (11% and 11%) and vegetables (15% and 17%) for foods with low-carbon footprint (37% and 42%). The percentages listed above in parentheses are calculated on the basis of total sales for each period and reported for the first and second interventions, respectively.

Table 2 shows the proportion of high/medium-carbon and low-carbon item sales for both item placement conditions (top vs. bottom). Overall, results highlighted the proportion of high/medium-carbon and low-carbon sales was significantly different between two interventions (both *p* values < 0.001), highlighting that dining hall students were more likely to choose low-carbon options when these were placed at the top of the menu (second intervention) (OR = 1.22; 95% CI = 1.21, 1.23). The same effect of placement can be observed for items with a medium- to high-carbon footprint during the first intervention when they were placed at the top of the menu.

**Figure 3:**
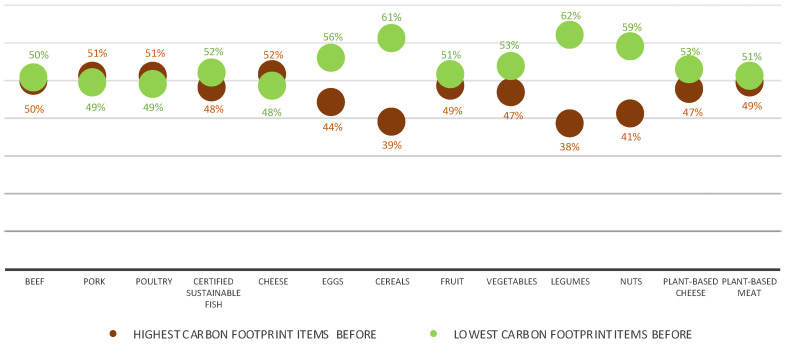
Distribution of total sale percentages for both intervention periods by each food category.

Figure 3 shows that the sales of all plant-based options significantly increased during the second intervention (i.e., low-carbon-footprint item at the top). Most notably, legumes, cereals, and nuts were the foods with the largest increase, followed by vegetables, plant-based cheese, and fruit. Among animal-based foods, eggs and certified sustainable fish options rose as well. Conversely, fewer sales were recorded for food items such as cheese, poultry, and pork when they were placed at the bottom. In addition, no significant changes were recorded for beef and plant-based meat options.

## 4. Discussion

The present study provides more evidence about the effects of strategic menu reordering to encourage the purchase of low-carbon-footprint options in university dining services. The placing of lower-carbon-impact options at the top of the menu (second intervention) resulted in higher sales as compared to when they were placed at the bottom of the menu (first intervention). At the same time, the purchase of higher-carbon-impact items, such as pork, poultry, and cheese, decreased when they were located at the end of the list. Unfortunately, the beef sales were not impacted by the menu reordering and remained stable even when listed at the end of the menu.

As noted in the literature, interest in using implicit interventions to drive people’s food choices has been growing rapidly in recent years [41,42,43,44]. Notably, several studies have analyzed the impact of menu item placement on customers’ food choices, showing mixed effects. Feldman and colleagues [45] suggested that people are more likely to select options positioned at the upper level of the menu because they are the most prominent, best remembered, and require less effort in the choice process, resulting in easier choices. Similar findings recently reported by Gynell et al. [46], confirmed that food placement can be an effective and promising strategy to encourage healthy eating choices, especially when implemented in an online menu. Consistent with our results, placing healthy snacks at the top of the menu resulted in a higher probability of being chosen by consumers than their placement in the middle or lower level of the menu. In addition, a large-scale field study evaluated the effect of four different nudging strategies (i.e., normative goal, hedonic goal, a combination of normative and hedonic influence, and menu placement) to promote the purchase of green foods category (i.e., vegetarian and vegan options) in fast-food restaurants. Placement of the green category at the top of the menu led to a significant increase in the number of vegetarian and vegan dishes chosen [47]. As confirmation of this, among the implicit techniques applicable to the food choice environment, menu re-ordering was found to be the most effective in guiding diners’ decision making towards low-environmental impact dietary selections [35].

However, not all studies about menu item placement have been consistent. Some studies have shown opposite results. Choi and colleagues [48] highlighted the tendency of consumers to focus their attention on the center of the menu and select options from that area, while Dayan and Bar-Hillel [49] pointed out that diners prefer lower items as much as upper ones because they are attracted to both extremes of the list. However, these mixed results reported in the literature may be related to several factors such as the number of items, menu layout, number of panels (e.g., one-fold, two-fold, etc.), and different study populations.

Beef sales trends in our study may have been affected by the same gaze pattern reported by Dayan and Bar-Hillel [50], who reported that items placed at the beginning or end of a list were more likely to be noticed. The failure of the intervention on beef options could also be related to the large intake of beef in the United States, emphasizing that people intentionally choose it regardless of its position on the menu, which makes it more difficult to discourage its consumption. In contrast, pork and poultry options were less popular during the second intervention, although both are widely consumed in the United States [51].

This study is an example of an implicit intervention to encourage low-carbon-impact food choices in dining service. Reordering the menu from the lowest to the most environmentally impactful item led to 22% higher odds of selecting a low-carbon option. This contrasted with placing the low-carbon-footprint foods below high-carbon food choices on the menu. This may be considered a small effect but considering the growth in the number of consumers eating outside of their homes [51], the spread of self-service kiosks in food services [52], and menu reordering being an intervention that is easy to deploy and sustain over time, its implementation could contribute significantly to reducing GHG emissions in the long run. Also, implicit nudge techniques, such as item placement, do not deter people from making a purchase, but rather can gently push them toward healthier behaviors and, for this reason, are more readily accepted by food suppliers and consumers [53]. In addition, human beings tend to influence each other in behaviors, including food choices. For instance, people are more likely to choose healthy foods when other consumers also choose them [54]. Therefore, the implicit promotion of more sustainable menus in a real-life setting, such as a university cafeteria, but also in other dining contexts, could have a positive impact on the clients and an exponential effect on the community [55].

However, it is important to point out the inherent limitations of this research. First, the study tested only the two opposite interventions (i.e., highest carbon at the top and lowest carbon at the bottom and vice versa) and not including a control data collection when the menu was in alphabetical order, as usual. Since the study was initiated immediately after the reopening of the closed dining location during the pandemic, we had no information available that could serve as baseline data. This may have amplified the effect of the results; nevertheless, the increase in sales for items located at the top of the menu was confirmed in both the first and the second intervention for the highest- and lowest-carbon items, respectively. Second, due to the study design that only relied on the recording of sales data, it was not possible to obtain further information about customers and possible predictors of food choice, nor was it possible to assess the nudge effect over time. In addition, recruiting a sample of students and evaluating the intervention through their choices would have avoided possible biases related to different sales numbers between the two interventions. Moreover, all the students were on a meal plan, and the price of items was not a determining factor. Overall, these aspects represent starting points for future studies aimed at deepening how the placement of dishes on the menu affects university students’ food choices.

## 5. Conclusions

Overall, results demonstrated a positive impact of menu re-ordering to increase climate-friendlier dietary choices and decrease consumption of foods with medium- to high-carbon-footprint values. However, the choice of beef options did not change as a function of menu reordering. This highlights the need for multiple strategies combining different types of interventions focused on raising students’ awareness of the environmental impact of animal-based foods, particularly beef. Current strategies at UCLA to increase students’ food literacy include university programs, seminars, Teaching Kitchen events, posters, and infographics with information about the carbon footprint of food in the dining halls, and high- and low-carbon-footprint icons added to online and in person menus. Given these promising findings from this study, menu re-ordering can be an easy approach to implement at university restaurants, and in catering services in general, to encourage the purchase of low-carbon-footprint menu options.

## Figures and Tables

**Table 1:** Distribution of the items sold expressed as the frequency of purchases by food category during the two interventions.

Food Category (*n* *)	First Intervention (*n* = 279,219)	Second Intervention (*n* = 288,527)	*p* Value
High-carbon footprint (*n* = 15)			
Beef (*n* = 2)	7208	7304	0.430
Cheese (*n* = 13)	73,312	70,712	<0.001
Medium-carbon footprint (*n* = 21)			
Pork (*n* = 15)	50,361	48,849	<0.001
Poultry (*n* = 6)	42,388	40,585	<0.001
Low-carbon footprint (*n* = 37)			
Eggs (*n* = 6)	11,536	14,624	<0.001
Plant-based meat (*n* = 3)	2797	2887	0.238
Certified sustainable fish (*n* = 2)	2645	2864	0.003
Plant-based cheese (*n* = 4)	3389	3746	<0.001
Legumes (*n* = 4)	4840	7794	<0.001
Nuts (*n* = 1)	3529	5037	<0.001
Cereals (*n* = 1)	1124	1768	<0.001
Fruit (*n* = 4)	31,098	32,956	<0.001
Vegetables (*n* = 12)	42,992	49,400	<0.001

Data are reported as absolute number. The binomial proportion test was applied by considering a *p*-value less than 0.05 as statistically significant. First intervention: highest-carbon-footprint items at the top and lowest at the bottom; second intervention: lowest-carbon-footprint items at the top and highest at the bottom. * *n* reflects number of items represented by the category.

**Table 2:** Distribution of total sales for food category (high/medium carbon vs. low carbon) by item placement condition.

Food Category	First Intervention (*n* = 279,219) *	Second Intervention (*n* = 288,527)	*p* Value
High/medium carbon	175,269 (51.1)	167,450 (48.9)	<0.001
Low carbon	103,950 (46.2)	121,077 (53.8)	<0.001

Data are reported as absolute number (% of total sales calculated by food category for each intervention period). The binomial proportion test was applied by considering a *p*-value less than 0.05 as statistically significant. First intervention: highest-carbon- footprint items at the top and lowest ones at the bottom; second intervention: lowest-carbon-footprint item at the top and highest at the bottom. * *n* reflects number of items sold for each intervention period.

## Data Availability

The data presented in this study are available upon reasonable request from the corresponding author (C.C.). The data are not publicly available.

## Supplementary Materials

The following supporting information can be downloaded at: https://www.mdpi.com/article/10.3390/nu15183873/s1, Supplementary Material S1: Configuration of menu options and customizable ingredients before the re-ordering intervention.
