# Supplementary material for: Increasing the Consumption of Environmentally Friendly Foods in a University Dining Hall Using Menu Item Placement
Source: Nutrients. 2023 Sep 6;15(18):3873. doi: 10.3390/nu15183873 (PMC10537694; doi:10.3390/nu15183873)

Supplementary Materials S1

Configuration of menu options and customizable ingredients before the re-ordering intervention

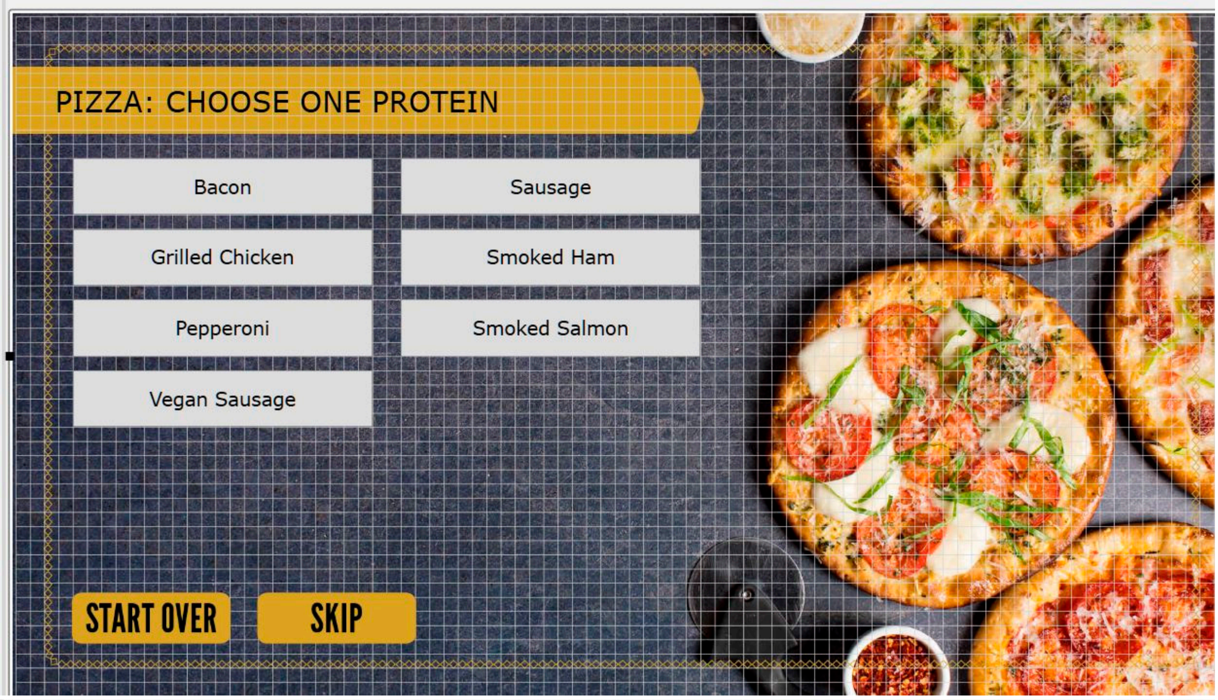

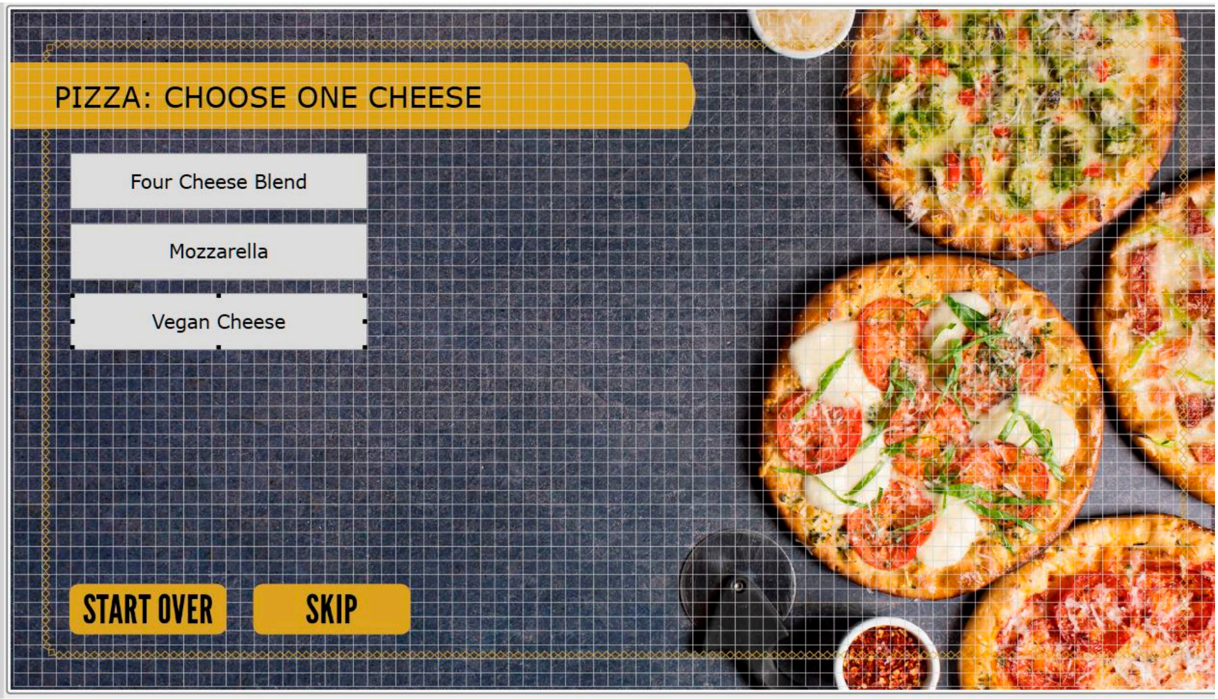

Supplementary Materials S1

Configuration of menu options and customizable ingredients before the re-ordering intervention

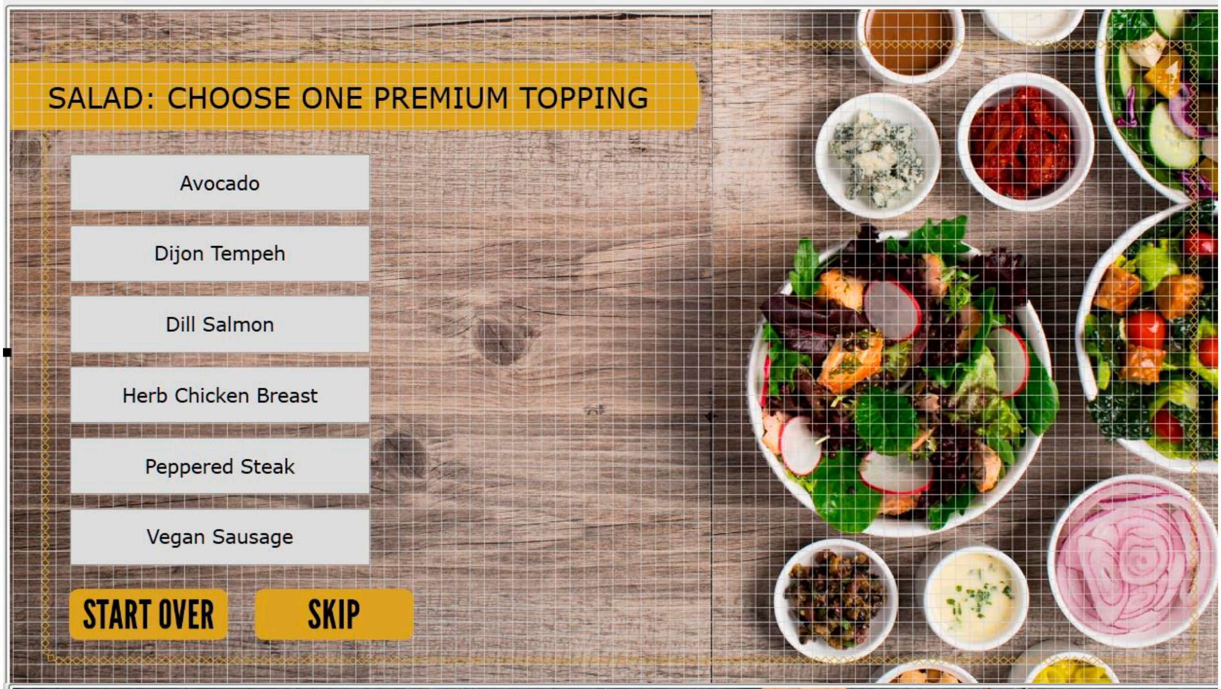

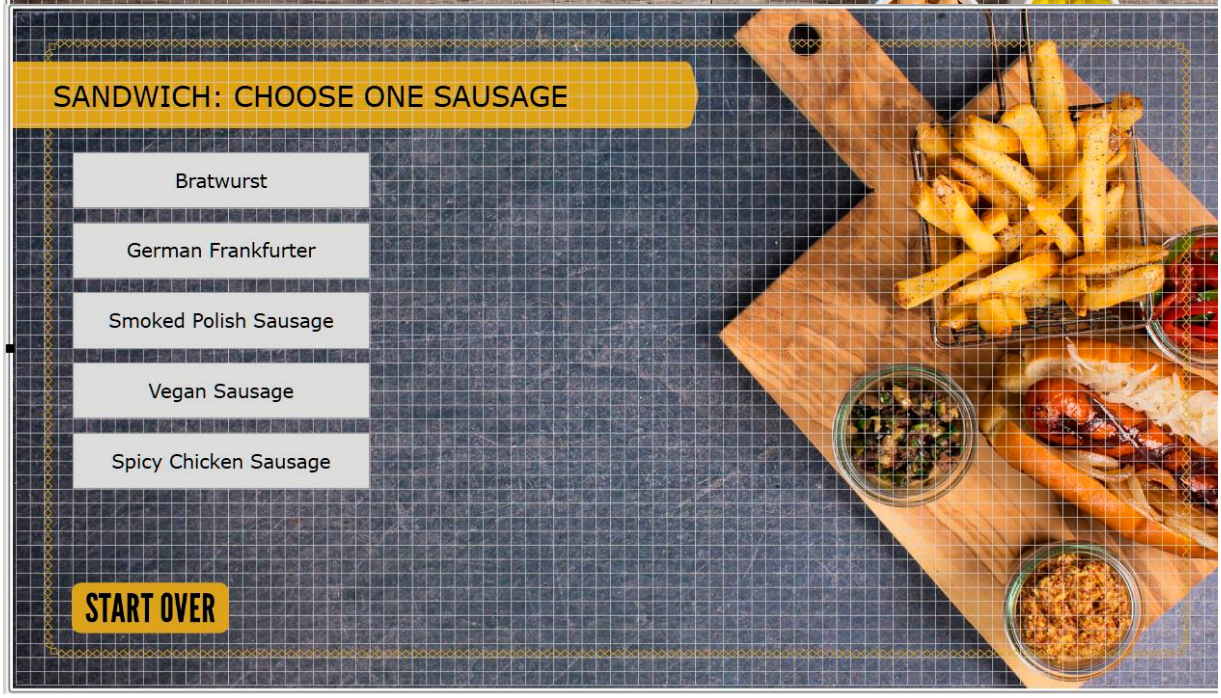

Supplementary Materials S1

Configuration of menu options and customizable ingredients before the re-ordering intervention

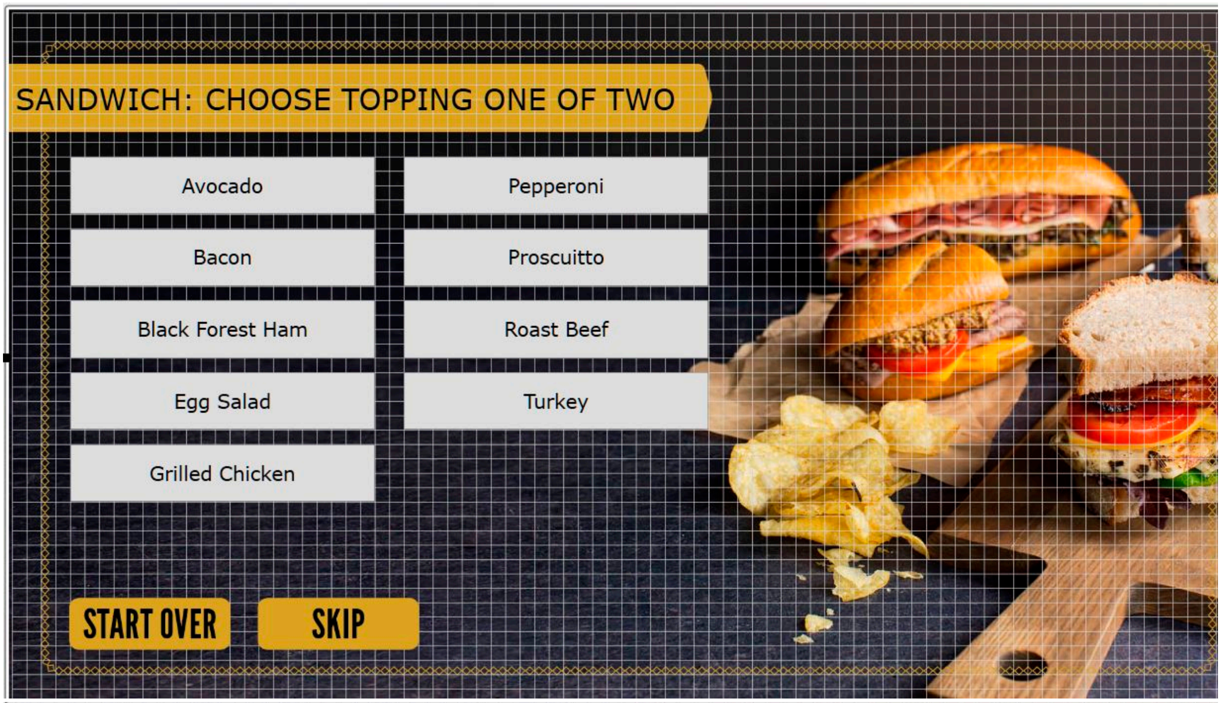

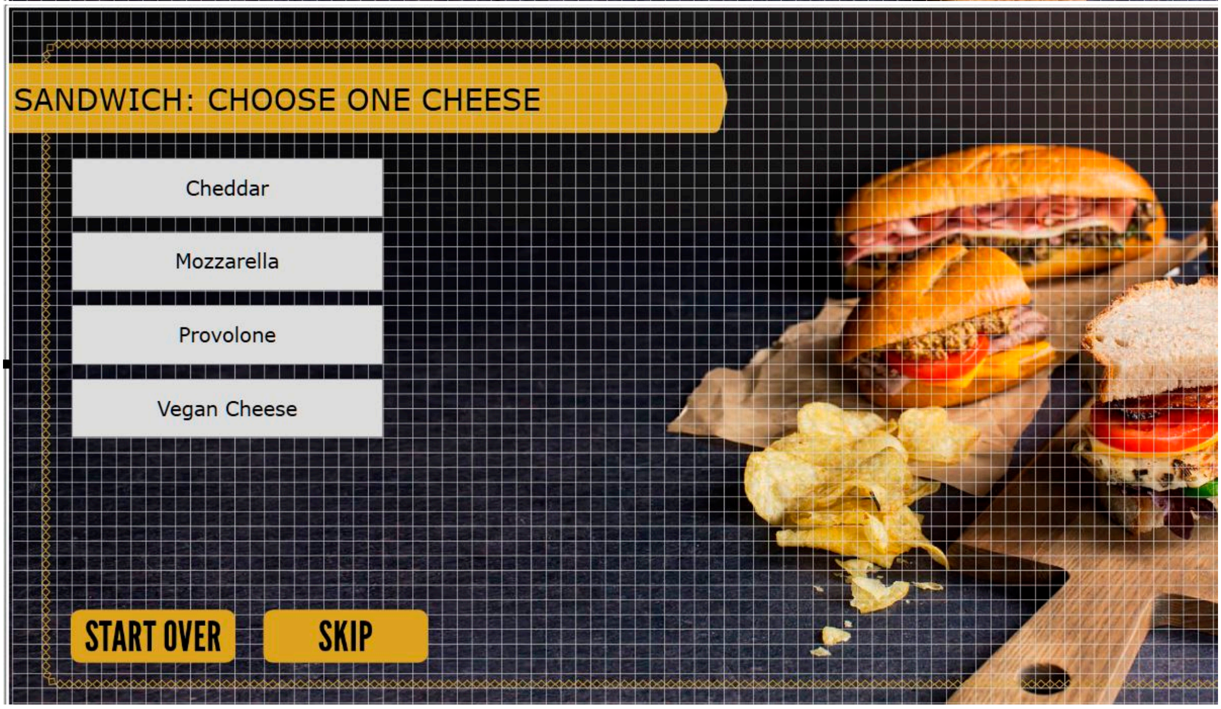

Supplementary Materials S1

Configuration of menu options and customizable ingredients before the re-ordering intervention

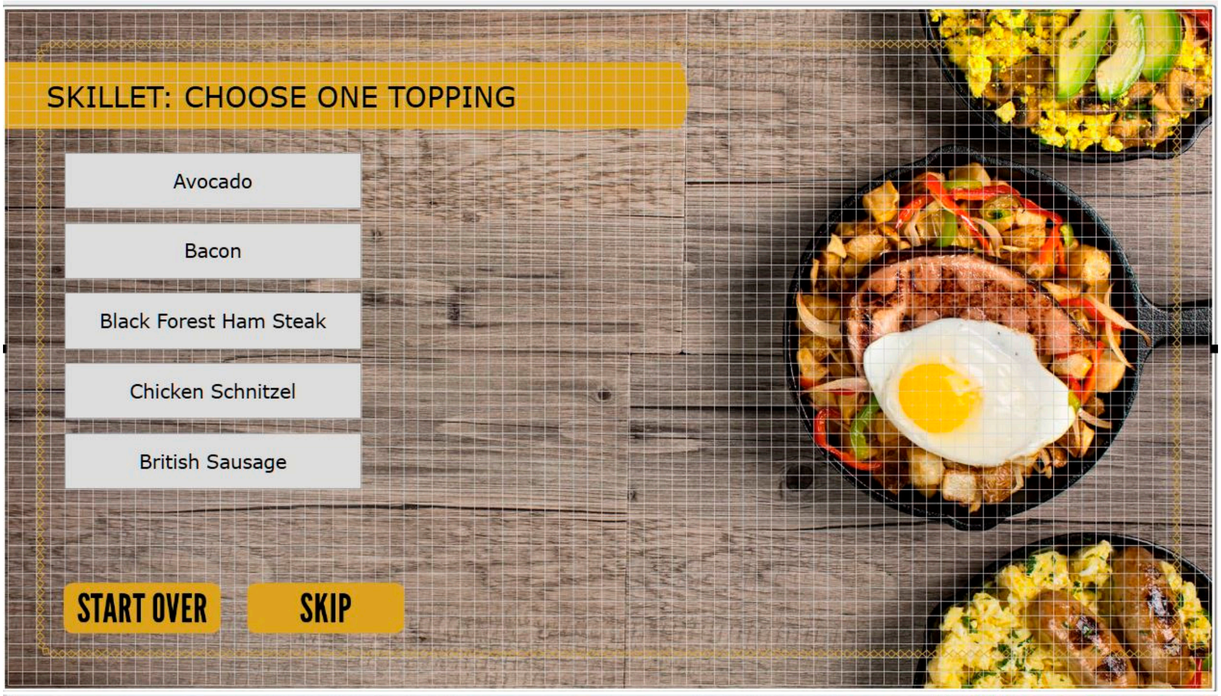

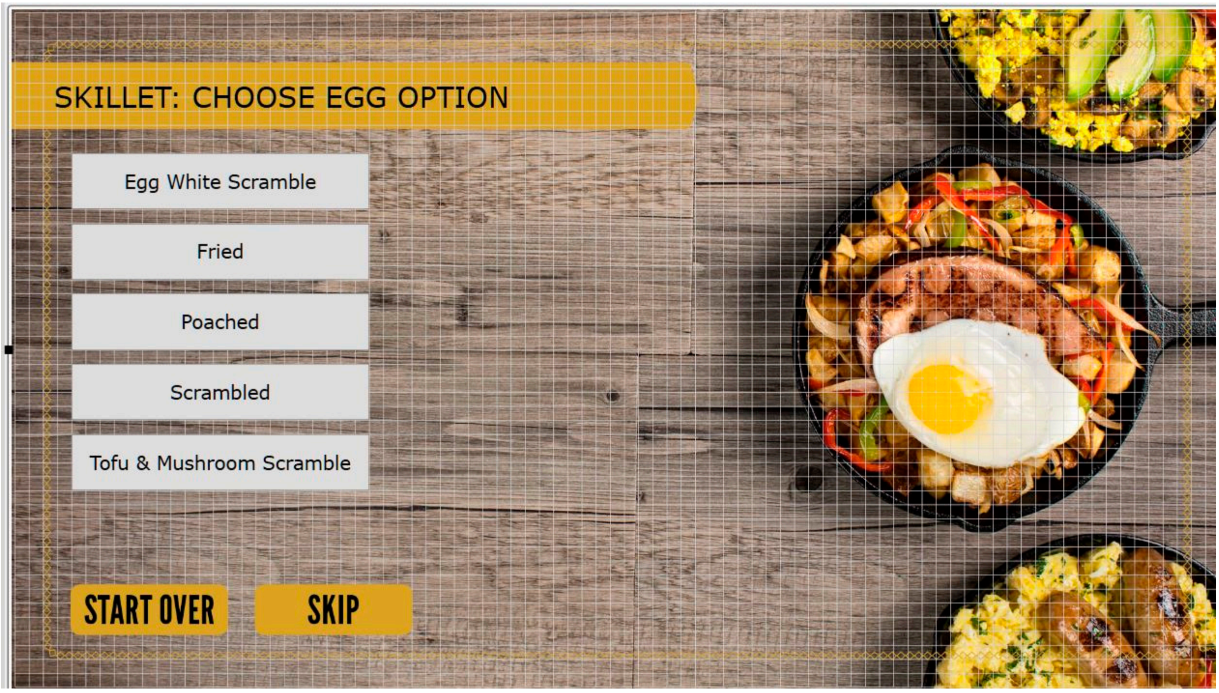

Supplementary Materials S1

Configuration of menu options and customizable ingredients before the re-ordering intervention

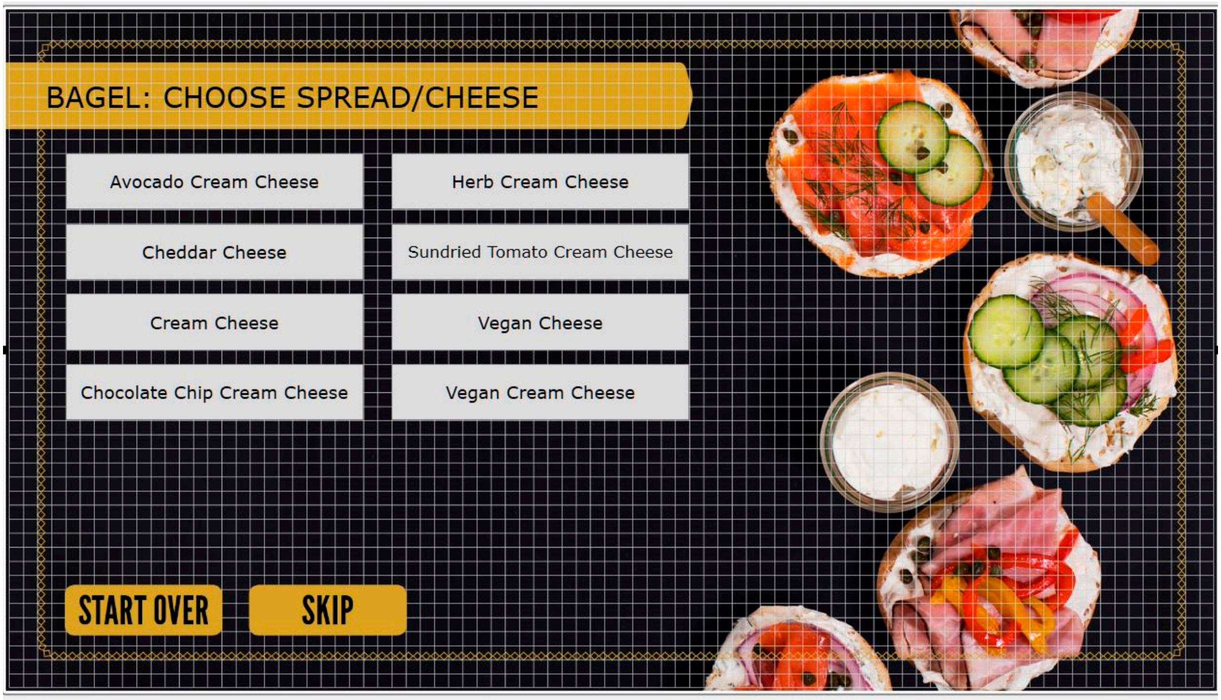

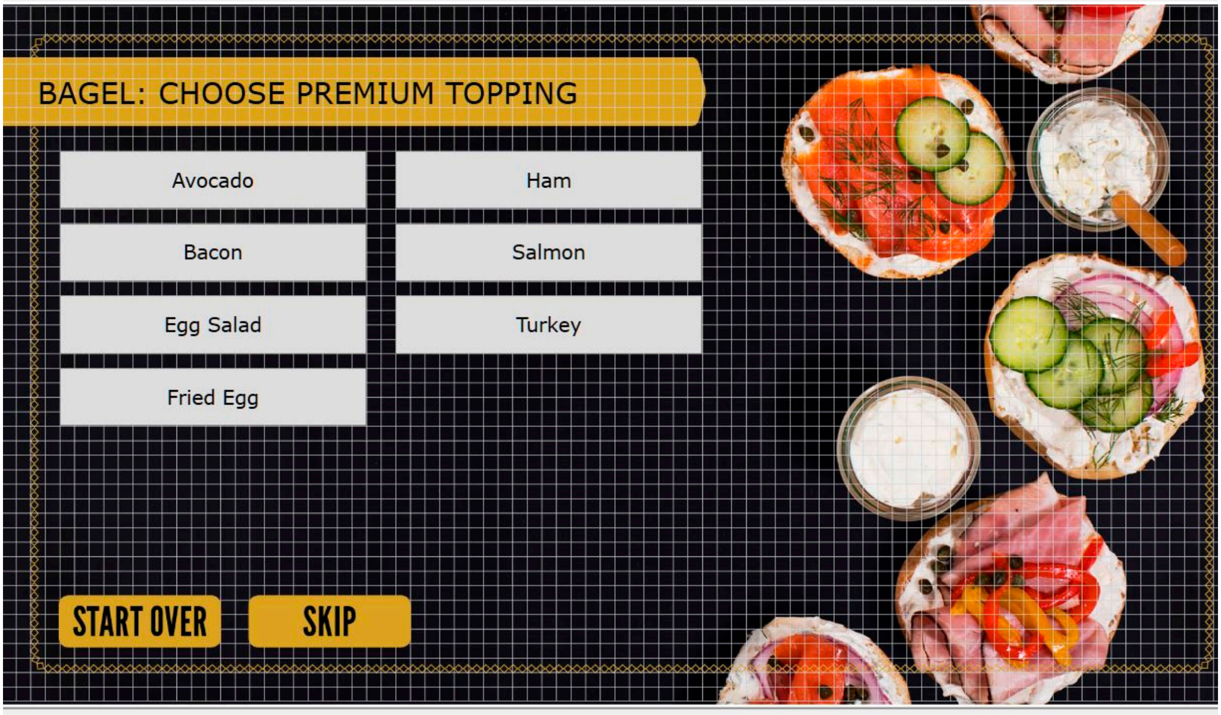

Supplement: Supplementary file 1 [file nutrients-15-03873-s001.zip › nutrients-2574202-supplementary.pdf]
